# The Pulse of AI: Implementation of Artificial Intelligence in Healthcare and its Potential Hazards

**DOI:** 10.2174/0118743064289936240115105057

**Published:** 2024-01-19

**Authors:** Syeda Farheen Zaidi, Asim Shaikh, Salim Surani

**Affiliations:** 1 Queen Mary University, London, E1 4NS, United Kingdom; 2 Department of Medicine, The Aga Khan University, Karachi 74800, Pakistan; 3 Department of Medicine & Pharmacology, Texas A & M University, College Station, Texas 77840, USA

**Keywords:** AI in healthcare, Bias, AI risks, Data inconsistencies, Healthcare disparities, AI privacy, AI in diagnostics, Democratization of AI, AI impact on healthcare professionals, AI framework, Patient data security

## Abstract

In this editorial, we explore the existing utilization of artificial intelligence (AI) within the healthcare industry, examining both its scope and potential harms if implemented and relied upon on a broader scale. Collaboration among corporations, government bodies, policymakers, and medical experts is essential to address potential concerns, ensuring smooth AI integration into healthcare systems.

Artificial intelligence (AI) refers to the application of technology to emulate critical thinking and intelligent behavior that is akin to a human being [1]. The term AI was coined by John McCarthy and further elaborated by Alan Turing who subsequently developed the Turing test. If an evaluator was unable to reliably distinguish machine responses from human responses, then the machine would have passed the Turing test. This work then led to further development in various AI domains [1, 2]. The utility of machine learning in biotechnology, pharmaceuticals, and the broader science, technology, engineering, and mathematics (STEM) domain has undeniably played a pivotal role in the advancements we are seeing today. On a molecular level, key think tanks have optimized their results by generative and computational AI, facilitating the replication and scaling of complex biochemical models [3]. Their models have been applicable in research drug delivery and have been applied in personalized patient therapies, such as in oncology [4]. Naturally, the healthcare industry is keen on unearthing AI's further potential. While the various benefits and capabilities of large-scale use of AI have been discussed in great detail, the potential hazards, which could be significant, have been overlooked in this context.

Let us begin with some important considerations. Introducing AI within a system is a highly resource-intensive feat. At the grassroots level, harnessing AI necessitates data mining and processing, employing machine learning algorithms, such as decision trees, boosting, and deep learning. Thereafter comes the cycle of testing, trials, troubleshooting, and overseeing the technology. This process will require a large investment of resources and may also disrupt the systems in place. Next, AI is as good as the data it has access to. For an AI model to integrate into a healthcare ecosystem, it will need to process an incomprehensible level of data. Unfortunately, electronic health records, registries, and biobank data are reasonably limited. The data are often riddled with inconsistencies, subjectivities, bias, and incorrect inputs by healthcare workers, leading to operator-dependent variability. Due to these factors, our little AI box of inaccuracies would thus be building its foundations on assumptions, correlations, and reductionisms it has learned, leading to counterintuitive results and spurious associations. To account for the gap in data, we need to explore the baseline disparities, find the drivers of disparities, elicit a bigger picture of bias, and establish ways to mitigate it.

Recently, AI-driven screening and surveillance have shown considerable promise, particularly in chronic diseases, such as diabetes. By creating multidimensional datasets through demographic and biochemical data, researchers have been able to establish predictive models with considerable accuracy [5]. However, it is essential to consider that medical technology has historically been designed with the Caucasian population in mind; therefore, some metrics are inaccurate for a broader, diverse population. While the algorithm keeps building upon the data it has, the screening and surveillance of underrepresented groups and how its conclusions may vary need to be taken into account. There is a possibility that AI could inadvertently lead to invasive testing or procedures, or conversely, dismiss critical health concerns. For example, African American women have a difficult time communicating their health concerns and feel ignored by healthcare professionals [6]. The AI model may thus hold the bias to simplify or overlook their concerns, potentially delaying intervention and causing a further breakdown of trust in the healthcare system.

Furthermore, one of the key determinants of AI implementation would be readiness and acceptability by healthcare workers. Many healthcare providers find themselves constrained by time to facilitate new changes as it may compromise their perceived quality of care. Therefore, it would require considerable effort for key individuals to convene and find a way to make the process work. Another important factor to consider is the accessibility of this system on a broader scale. Given that AI technology will only be available to a microscopic stratum of healthcare systems, the scenario arises where patients present from low-resource areas with limited data; the AI will then be completely unreliable and, if deeply integrated within the system, problematic to override. This is why the democratization of AI will be very relevant here, wherein, developer tools, libraries, and data sets would be made accessible so that AI can be easily developed in underrepresented areas [7, 8].

In certain scenarios, we must contemplate the risks associated with reliance on AI in healthcare. We have seen AI’s capabilities and intelligence advance at a rapid pace, from successfully passing the United States Licensing exams [9] to efficiently executing hospital administrative tasks, as seen with AI systems, like BotMD [10]. Perhaps, the most significant growth of AI is seen in radiomics and pathomics. Radiomics and pathomics are quantitative approaches to medical imaging analysis. Through deep learning techniques and feature engineering, the AI model assimilates a vast dataset of histomorphometry, intensities, colors, structures, textures, and spatiality, providing high levels of interrogation and computer visual processing. It is now very plausible to assume that AI can deliver both quantitative and qualitative findings with extreme levels of accuracy in its interpretations and may be able to detect minuscule changes better than human capabilities [11, 12].

A study demonstrated that AI was able to diagnose colorectal cancer, lung cancer, and liver cirrhosis more accurately than a board-certified pathologist, with 98% accuracy compared to 96.9% accuracy, respectively [13]. Similarly, AI predictions for assessing breast cancer risk have proven superior to a radiologist [14]. There is also a growing influence of AI in diagnostics. Through convolutional neural networks, an AI model learned to diagnose Kawasaki disease [15], which has traditionally posed diagnostic challenges. While these capabilities are useful, we must consider the impact they may have on healthcare professionals. The utility of AI can either lead to a strong reliance on this machine as a tool and degradation of expertise or, in a dystopian scenario, lead to a fundamental shift in roles, particularly in fields, such as radiology and pathology, where there is less patient interaction. Suppose AI-generated findings were established as superior for accuracy in reporting, in that case, it may engender diagnostic conflicts wherein healthcare professionals could find themselves struggling to challenge incorrect AI findings and may fear litigation in case of opposition.

Finally, one of AI's main vulnerabilities lies in privacy and data breaches. Considering the political climate and frequent penetrations of even the most robust strongholds of society by cyber-attacks, the healthcare industry may prove to be an easier target in comparison. Patient records would then be susceptible to distortion or leaks, and intelligent data could be sold to gain profits insidiously. Such scenarios are highly plausible and may result in an overall shutdown of the entire infrastructure, further incurring a hefty cost burden on the industry. In 2018, Google acquired DeepMind, a prominent player in healthcare AI. The National Health System of the United Kingdom transferred data from 1.6 million patients onto DeepMind servers without obtaining formal consent for AI research. This patient data was then utilized to develop Streams, an application that featured predictive algorithms for acute kidney injury; while good intentioned, it breached European data laws and was scrapped quickly by Google post its criticism [16, 17]. This is just one example of how sensitive patient data can be siphoned off to third parties.

The more popular concerns in media currently present doomsday scenarios with AI. Some philosophical threats about sentient AI intelligence explosion, self-governance, and instrumental convergence lead to a future akin to sky-net level AI [18]. Such claims are admittedly far-fetched and do not warrant a realistic concern. Nevertheless, legitimate debate about the appropriate application of AI and what it means for the healthcare industry is highly valid. A robust framework needs to be established to ensure the accuracy and reliability of AI-driven assessments. Some examples of regulatory features in the AI framework have already been proposed, such as ethical governance, which tackles fairness, privacy, and transparency. Explainability and interpretability are required to ensure comprehension of algorithmic decisions on a layperson level so that AI findings can be challenged. Finally, ethical auditing would examine the inputs and outputs of algorithms to identify any prevalent biases or harms in the system [19-21]. These features can be put into practice through technical approaches, such as algorithmic impact assessments, improving client-side data encryption, and wider AI education and training [16, 21]. Corporations, government bodies, and policymakers, all need to be a part of the broader discussion, with medical experts leading the charge for framework development and addressing potential concerns with AI implementation in healthcare systems.
